# Supplementary material for: Betanin, a Natural Food Additive: Stability, Bioavailability, Antioxidant and Preservative Ability Assessments
Source: Molecules. 2019 Jan 28;24(3):458. doi: 10.3390/molecules24030458 (PMC6384587; doi:10.3390/molecules24030458)
Supplement: Supplementary file 1 [file molecules-24-00458-s001.pdf]

Supplementary Materials

Betanin, a Natural Food Additive: Stability, Bioavailability, Antioxidant and Preservative Ability Assessments

Davi Vieira Teixeira da Silva, Diego dos Santos Baião, Fabrício de Oliveira Silva, Genilton Alves, Daniel Perrone, Eduardo Mere Del Aguila and Vania M. Flosi Paschoalin *

Instituto de Química, Universidade Federal do Rio de Janeiro, Av. Athos da Silveira Ramos 149, 21941-909 Rio de Janeiro, Brazil. daviufrj@outlook.com (D.V.T.d.S.); diegobaiao20@hotmail.com (D.d.S.B.); silvafo@live.com (F.d.O.S.); geniltonalves@gmail.com (G.A.); perrone@iq.ufrj.br (D.P.); emda@iq.ufrj.br (E.M.D.A.)

* Correspondence: paschv@iq.ufrj.br; Tel: +55-21-3938-7362; Fax: +55-21-3938-7266

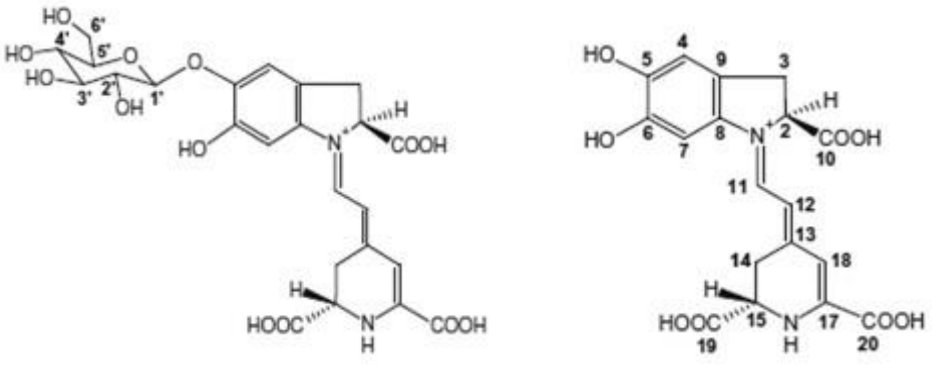

Figure 1. Chemical structure of betanin (A) and betanid in (B);.

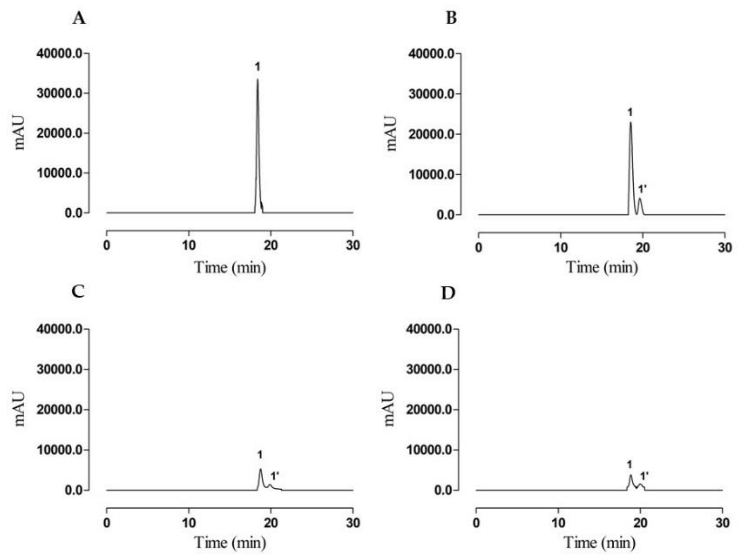

Figure 2. Betanin chromatograms before and after each in vitro digestion phase assessed by RP-HPLC equipped with DAD detector (536 nm). Purified betanin (A), after oral digestion (B), after gastric digestion (C), after small intestine digestion (D);.

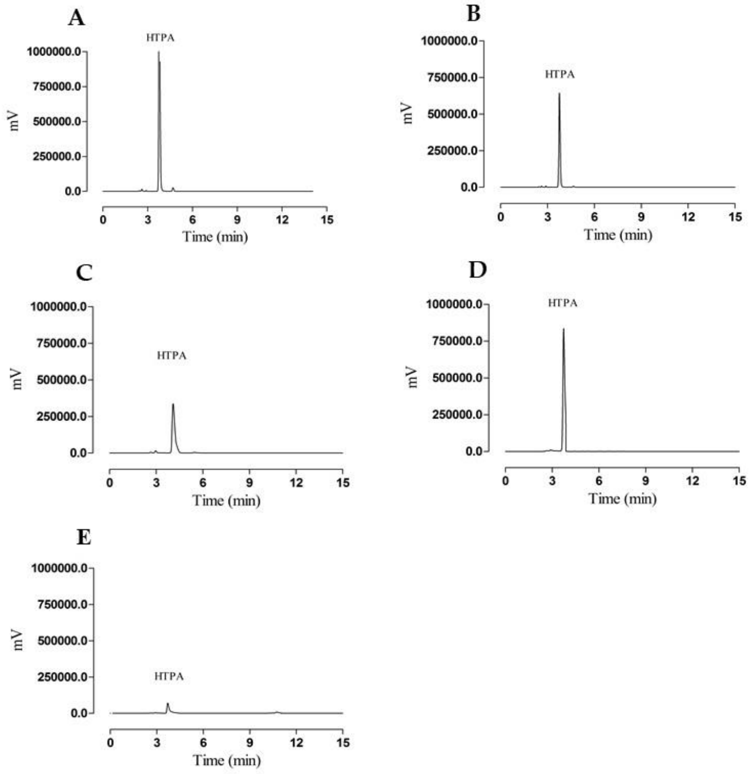

Figure S3. Betanin total antioxidant potential (TAP) assessed by RP-HPLC equipped with a fluorescence detector (312/428 nm). Hydroxyterephthalic acid (HTPA) chromatograms of generated in the Fenton reaction without any sample (A), after betanin addition (B), after oral digestion (C), after gastric digestion (D), after small intestine digestion (E);.

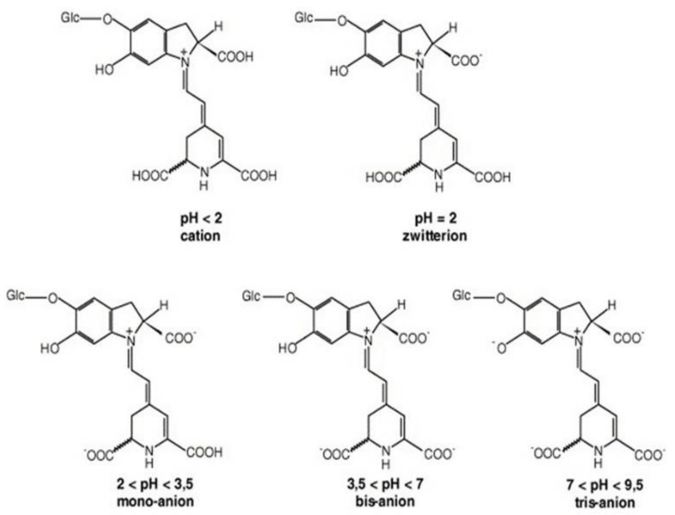

Figure 4. Influence of pH on betanin chemical structure charge changes in an aqueous solution according to Frank et al. [35].

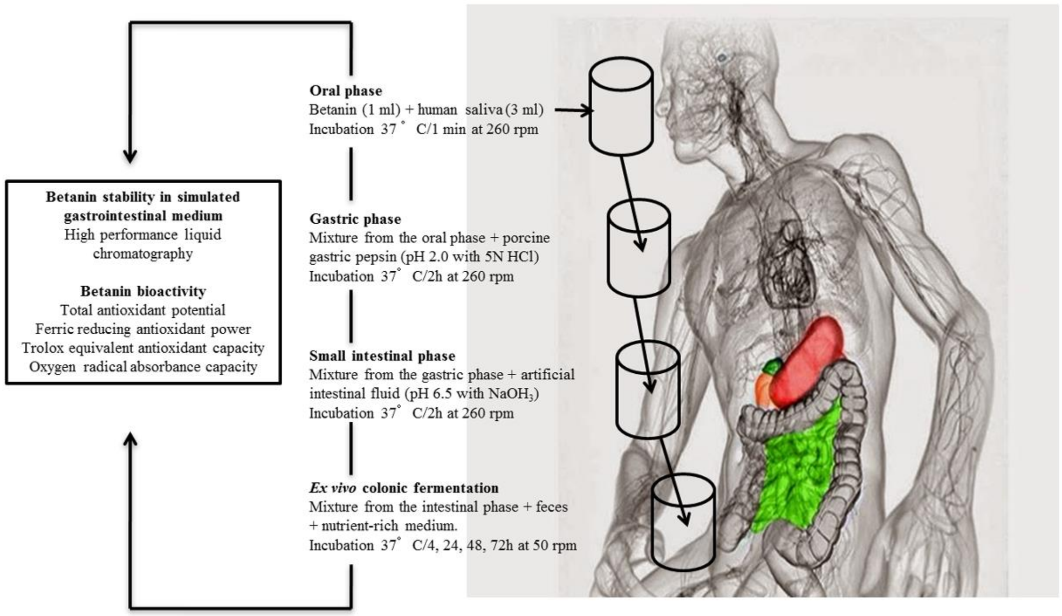

Figure S5. Simulated digestion scheme.
